# Motivational pathways from perceived teacher support to student engagement in EFL classes

**DOI:** 10.3389/fpsyg.2025.1677994

**Published:** 2025-11-20

**Authors:** Xiaohui Zhang, Hyun-Ju Kim

**Affiliations:** 1 Department of Foreign Languages, Shangqiu Normal University, Shangqiu, China; 2 Department of British and American Humanities, Dankook University, Gyeonggi-do, Republic of Korea

**Keywords:** teacher support, academic self-efficacy, achievement goal orientation, student engagement, EFL reading class

## Abstract

This study investigates the psychological mechanisms through which perceived teacher support influences student engagement in English as a Foreign Language (EFL) reading classes at Chinese universities. Drawing on social support theory and expectancy-value theory, the study examines how teacher support predicts students' motivational beliefs (self-efficacy and intrinsic goal orientation), and how these beliefs, in turn, mediate the link between teacher support and behavioral, emotional, and cognitive engagement. A total of 524 undergraduate EFL learners participated in the study. Data were analyzed using structural equation modeling (SEM), which confirmed the hypothesized motivational mediation pathways. The findings indicate that perceived teacher support significantly predicts these two motivational beliefs, which subsequently lead to higher levels of behavioral, emotional, and cognitive engagement. The results provide theoretical implications for understanding engagement through established psychological constructs and practical guidance for fostering motivation in language learning settings. Overall, this study contributes to educational psychology by applying motivational theories to second language learning contexts and providing evidence-based insights on how to foster student engagement through both contextual and psychological mechanisms.

## 1 Introduction

As transformative agents in educational ecosystems, teachers play a pivotal role in EFL reading classrooms, where their strategic support not only fosters immediate student engagement but also signifies their ongoing professional growth (Belmont et al., 1992; Bozkurt, 2022; Fredricks et al., 2004; Roorda et al., 2011). Such support is most effective when it triggers essential cognitive and motivational mechanisms in learners, ultimately leading to active and sustained reading engagement (Davis et al., 2018; Hospel and Galand, 2016; Pitzer and Skinner, 2017; Ryan and Deci, 2017).

Student engagement in English reading involves active cognitive and metacognitive processes, underpinned by intrinsic motivation and reinforced through an awareness of both immediate instrumental utility and long-term usefulness of extensive reading with informational texts (Barber et al., 2016; Guthrie and Klauda, 2014; Wigfield and Guthrie, 2000). It shapes learners' motivation, fosters language proficiency, and contributes to long-term mastery of English (Cooper et al., 2014; Protacio, 2017; Yang et al., 2024). Despite its importance, the roles of teacher support and student engagement have not received sufficient attention in English as a Foreign Language (EFL) reading and applied linguistics research (Sadoughi and Hejazi, 2021). Recent investigations in China point to declining levels of engagement, motivation, and satisfaction among English learners. Teachers often report minimal student participation, while learners themselves express concerns about the limited value they perceive in classroom instruction (Mao, 2021; Wang, 2017). These issues are compounded by insufficient consideration of learners' basic psychological needs and a lack of positive motivational beliefs, such as strong self-efficacy and adaptive goal orientations.

To support students in overcoming these challenges, EFL teachers should provide targeted pedagogical support to help students read and stay engaged in this endeavor (Finkbeiner et al., 2016). Student engagement in reading emerges from the dynamic interplay between contextual and personal factors, both of which collectively shape reading practices and learning outcomes (Woreta, 2024). Effective teaching strategies are vital in cultivating such an atmosphere. Educators play a crucial role in supporting key psychological needs that drive motivation and maximize reading engagement. According to Self-Determination Theory (Deci and Ryan, 1985), fulfilling students' fundamental psychological needs can promote their intrinsic motivation and self-regulated learning. Teachers can nurture these needs by offering choices in reading activities, showing care for students, and providing clear and constructive feedback. Simultaneously, Social Cognitive Theory highlights the role of academic self-efficacy as a core predictor of learning behavior and persistence (Bandura, 1977). Additionally, Achievement Goal Theory shapes how learners approach academic tasks. Mastery goals are associated with deeper learning and persistence, while performance-avoidance goals often correlate with anxiety and disengagement (Elliot and McGregor, 2001). Academic self-efficacy and goal orientations—key motivational beliefs—thus reflect students' self-perceptions about competence and expected outcomes in reading, which strongly influence engagement (Collie and Martin, 2019). Importantly, teacher support, student engagement, and achievement goal orientation are not monolithic variables but multidimensional constructs. Each dimension—for example, autonomy, competence, and relatedness support on the teacher side, or behavioral, cognitive, and emotional aspects of engagement on the student side—may function differently and exert distinct influences. Acknowledging these multiple dimensions is essential for understanding the mechanisms and boundary conditions of motivational processes in EFL reading contexts.

Previous studies have shown that need-supportive teaching approaches can foster stronger motivation, greater resilience, and improved academic outcomes (Jang et al., 2016; Pitzer and Skinner, 2017). Yet, there is still a lack of empirical research that integrates self-determination theory, academic self-efficacy, and achievement goal orientations into a unified framework to explain how teacher support mediates EFL reading engagement. Moreover, while engagement has been widely studied in STEM fields (e.g., Alrajeh and Shindel, 2020; Strati et al., 2017), relatively little attention has been paid to English reading (Eren and Rakicioglu-Söylemez, 2021). To address this gap, this study explicitly examines both the overall construct-level relationships and the dimension-level effects of teacher support, student engagement, and motivational beliefs. This dual approach not only clarifies the general pathways but also allows for a closer inspection of how individual dimensions contribute to the mediation and moderation processes. In doing so, the study aligns the multidimensional nature of the constructs with its central focus on motivational pathways in EFL reading.

Building on this perspective, the present study explores how teacher-provided autonomy, relatedness, and competence support shape student engagement in Chinese university EFL reading classrooms. Specifically, it further examines the mediating role of academic self-efficacy and the moderating role of achievement goal orientations. By mapping the motivational pathways underlying student engagement, this research seeks to contribute practical insights for instructional design and educational policy within EFL contexts. Accordingly, the study poses the following research questions:

Research Question 1: How does teacher support predict Chinese university students' engagement in EFL reading classes?
Research Question 2: How does students' personal motivational belief—academic self-efficacy—mediate the relationship between teacher support and student engagement in EFL reading classes?
Research Question 3: How does students' personal motivational belief—achievement goal orientation—moderate the relationship between teacher support and student engagement in EFL reading classes?

## 2 Literature review

### 2.1 Student engagement

Widely regarded as a multifaceted or meta-construct, student engagement has emerged as a central concern in both educational and developmental psychology (Guthrie et al., 2012; Wolters and Taylor, 2012). It generally refers to the active involvement and commitment in learning tasks. Engagement is typically conceptualized as comprising behavioral, cognitive, and emotional dimensions (Fredricks et al., 2004). Behavioral engagement includes participation and persistence, cognitive engagement involves mental effort and strategy use, and emotional engagement reflects affective reactions such as interest or anxiety (Guthrie et al., 2012). While these dimensions are universal, their manifestations may differ across contexts (Janosz, 2012). Empirical studies consistently show that engagement predicts academic outcomes. Longitudinal research links higher engagement to achievement and reduced dropout intentions (Alivernini and Lucidi, 2011), and meta-analytic findings confirm its role in persistence and success (Roorda et al., 2011; Wentzel et al., 2016). In reading, validated engagement measures predict intervention gains (Martinez-Lincoln et al., 2021) and classroom activity profiles associated with sustained participation (Barber et al., 2016). In EFL contexts, engagement is shaped by individual motivation and instructional design, with engaged readers demonstrating stronger strategy use and persistence (Protacio, 2017). Recent studies also suggest that technology-enhanced instruction, such as online platforms and interactive exercises, can strengthen reading motivation and engagement (Gao, 2023). In line with this literature, we operationalize student engagement at both the overall construct level and by its three dimensions (behavioral, cognitive, emotional) to examine whether associations differ across facets.

### 2.2 Teacher support based on self-determination theory

Self-Determination Theory (SDT; Deci and Ryan, 1985), offers a comprehensive framework for examining motivation in second language (L2) learning, and its value has been increasingly validated through empirical research (De Smedt et al., 2020). Within this framework, motivation is conceptualized along a continuum ranging from amotivation to extrinsic and intrinsic motivation, representing varying degrees of autonomy (Cook and Artino, 2016). A central process in SDT is internalization, where external expectations are gradually adopted as personal values, and integration, where these values become part of one's identity, identifying the higher degree of self-determination (Cook and Artino, 2016).

One of the SDT's sub-theories, Basic Psychological Needs Theory, posits that intrinsic motivation is nurtured when the social context fulfills three innate psychological needs: autonomy (the sense of volition and self-direction), relatedness (feeling connected to others), and competence (a sense of capability and effectiveness) (Ryan and Deci, 2017). Autonomy-supportive contexts minimize external control, fostering volitional engagement (Han, 2021). Relatedness-supportive contexts emphasize care, respect, and emotional understanding (Kaefer and Chiviacowsky, 2021). Competence-supportive contexts provide clear goals, guidance, and feedback (Jackson-Kersey and Spray, 2016). Blocking of any of these three basic needs undermines students' autonomous motivation (Ryan and Deci, 2020). Conversely, when the school context fulfills these needs, students display stronger autonomous motivation, adaptive learning behaviors, and improved academic outcomes (Núñez and León, 2019).

A substantial body of empirical evidence supports these claims. Autonomy support has been linked to reduced fatigue and heightened emotional engagement (Montenegro and Schmidt, 2023) as well as stronger motivation in Japanese EFL extensive reading (Tanaka, 2017). Relatedness support predicts self-regulated learning behaviors such as metacognitive monitoring and persistence (Schuitema et al., 2016), while competence support fosters academic values and effort (Wentzel et al., 2016). Studies in classroom contexts further demonstrate that autonomy-supportive teaching and instructional structure together promote higher engagement and adaptive outcomes (Hospel and Galand, 2016; Jang et al., 2016). More recent evidence from East Asian EFL settings also indicates that teacher support enhances students' reading persistence, comprehension, and positive attitudes (Finkbeiner et al., 2016; Han, 2021), highlighting the critical role of teachers in cultivating motivation and engagement across diverse cultural contexts.

### 2.3 Motivational beliefs

Motivational beliefs have been widely conceptualized through two well-established and validated frameworks: academic self-efficacy, derived from Social Cognitive Theory (SCT), and achievement goal orientation, derived from Achievement Goal Theory (AGT). These frameworks explain students' motivated behaviors from complementary perspectives—competence judgment and goal pursuit. Building on the integrative framework proposed by Pintrich (2003), the present study focuses on these two central constructs to examine how motivational beliefs operate in EFL contexts.

Self-efficacy (SE) refers to individuals' belief in their capability to accomplish a given task successfully (Bandura, 1986). It shapes learners' emotion, motivation, and action (Çikrikci, 2017). Students with high self-efficacy are likely to perceive challenging tasks as opportunities for growth rather than as barriers (Zysberg and Schwabsky, 2021), which encourage deeper involvement, higher achievement, and better psychological adjustment. A substantial body of evidence indicates that self-efficacy predicts persistence, strategy use, and attainment in reading (Lam et al., 2012; Waleff, 2010; Templin, 2011), and recent findings with Chinese university students show that reading self-efficacy strongly contributes to enjoyment, engagement, and performance (Yang et al., 2024). Within SCT's triadic reciprocal model, academic self-efficacy interacts with behavioral and environmental factors, particularly teacher support, which strengthens efficacy through autonomy, relatedness, and competence support (Roorda et al., 2011; Trouilloud et al., 2006). Across domains such as mathematics, science, and reading, self-efficacy consistently emerges as a robust predictor of effort and academic outcomes (Schunk and Pajares, 2009; Lam et al., 2012).

Achievement Goal Orientation Theory (AGO) explains individuals' task approach and goal pursuit (Ames, 1992). The framework has evolved from mastery vs. performance goals (Dweck and Leggett, 1988) to a trichotomous model distinguishing performance-approach and performance-avoidance (Middleton and Midgley, 1997), and finally to the 2 × 2 model applying approach-avoidance distinctions to mastery goals (Elliot and McGregor, 2001). Goals inherently function as powerful motivators, and external factors influence behavioral drive and performance through one's perception of those goals. Thus, when encountering the same task, individuals with a strong goal orientation—because they perceive the goal as both attainable and valuable—tend to possess a clearer sense of direction and purpose. As a result, they are more inclined to invest greater effort and demonstrate deeper engagement in the activity (Jeong et al., 2023). In addition, empirical studies suggest that different forms of goal orientation can either decrease or increase students' propensity to procrastinate and their degree of academic engagement (Muñoz-Olano and Hurtado-Parrado, 2017). Among struggling readers, mastery and performance-avoidance goals moderate the relationship between mindset and reading proficiency via engagement (Cho et al., 2019). In EFL contexts, learners' orientations likewise shape study behaviors and reading strategies (Shyr et al., 2017), underscoring the continuing relevance of AGO to L2 reading engagement.

### 2.4 Theoretical and conceptual framework

As outlined in the preceding sections, teacher support and personal motivational beliefs (academic self-efficacy and achievement goal orientation) are core variables for understanding students' learning engagement and learning process. These variables exist within a dynamic and interactive system. To unravel the intrinsic relationships and pathways of influence among them, this study integrates these theories to provide a comprehensive explanatory model.

This study applies SDT as the overarching theoretical foundation, highlighting the central role of teacher support as an environmental driver of motivation. Within this framework, teacher support fosters intrinsic motivation and more autonomous forms of extrinsic motivation by satisfying students' basic psychological needs. In doing so, it functions as the contextual force that initiates and sustains the motivation–engagement process.

**Figure 1:**
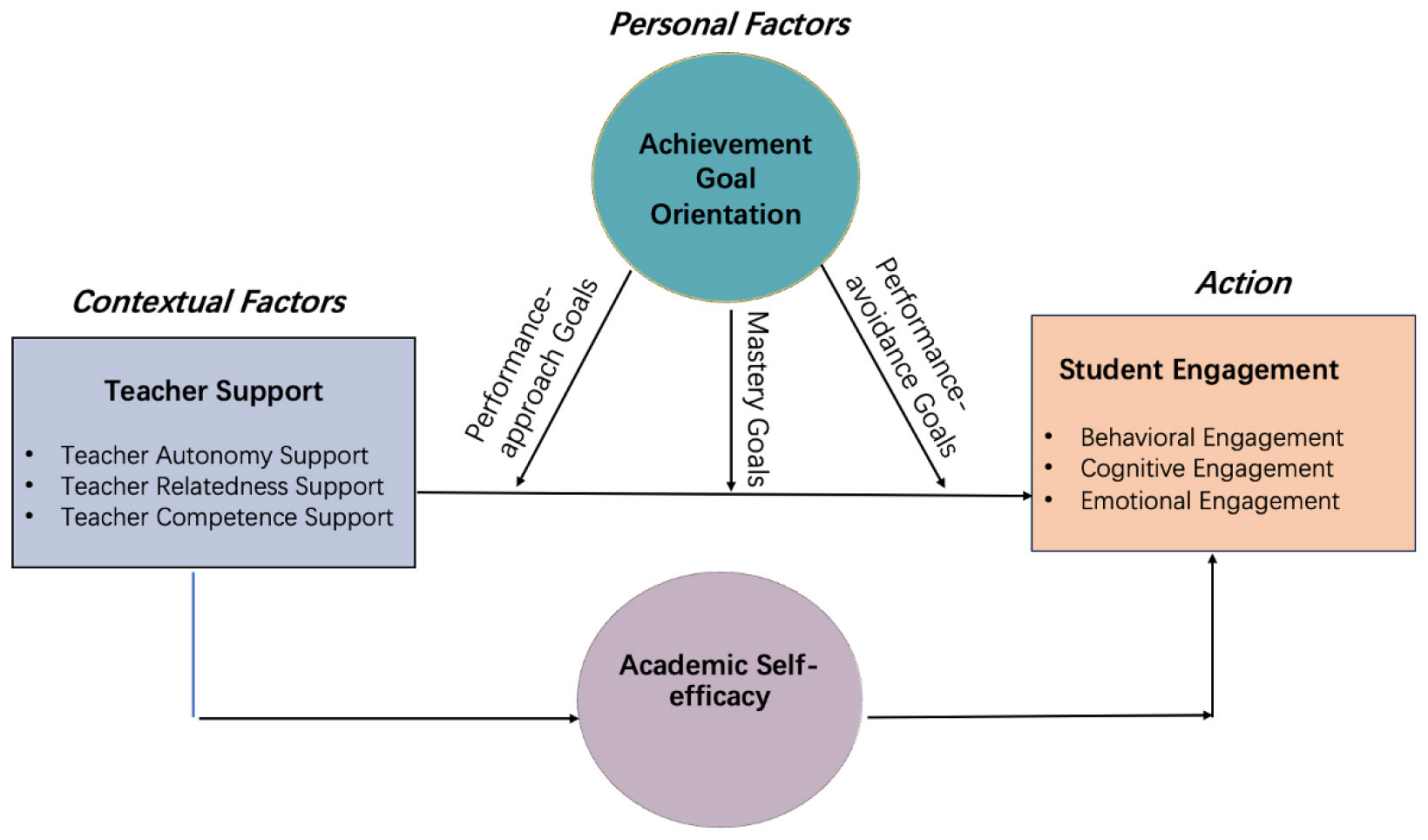
Conceptual research framework.

Personal motivational beliefs are introduced as mediating and moderating mechanisms to explain how the effect occurs. While SDT addresses why teacher support is effective, the question of how—that is, through which psychological processes teacher support translates into student engagement—requires further elaboration. To this end, academic self-efficacy is incorporated as the mediator and achievement goal orientation as the moderator. Figure 1 presents the conceptual framework of this study.

### 2.5 Research hypotheses

Based on the integrated framework, this study proposes the following hypotheses to examine the underlying mechanisms linking teacher support, motivational beliefs, and student engagement. According to SDT, teacher support enhances intrinsic and autonomous motivation, leading to more active and persistent engagement (Jang et al., 2016; Pitzer and Skinner, 2017). Therefore, we hypothesize:

**H1: Teacher support will positively predict student engagement**.

Drawing on SCT, academic self-efficacy is expected to function as a mediator between contextual support and engagement (Schunk and DiBenedetto, 2020). Thus, we hypothesize:

**H2: Academic self-efficacy will mediate the relationship between teacher support and student engagement**.

AGT suggests that mastery goals foster deeper engagement, while performance-avoidance goals undermine it (Collie and Martin, 2019; Elliot and McGregor, 2001). Thus, achievement goal orientation is expected to moderate the effect of teacher support.

**H3: Achievement goal orientation will moderate the relationship between teacher support and student engagement**.

These hypotheses correspond directly to the research questions introduced in Section 1.

Given that teacher support, student engagement, and achievement goal orientation are multidimensional constructs, with each facet potentially exhibiting distinct psychological and behavioural attributes, this study explicitly examines both the overall construct-level relationships and the dimension-level effects. This dual-level approach not only clarifies the general pathways among the variables but also allows for closer inspection of how individual dimensions uniquely contribute to the mediation and moderation processes. By incorporating both perspectives, the study provides a more comprehensive understanding of the motivational pathways.

## 3 Methodology

This study was conducted in accordance with the ethical principles of educational research. The Institutional Review Board of Dankook University approved the protocol as exempt from full review due to its minimal-risk nature and voluntary participation. Prior to data collection, informed consent was obtained from all participants, who were notified of the study's purpose, the voluntary nature of their participation, their right to withdraw at any time, and confidentiality safeguards.

### 3.1 Participants

The participants in this study were 524 Chinese college English majors, including 78 males (14.89%) and 446 females (85.11%). The sample was evenly distributed across academic years, with 131 freshmen (25.00%), 132 sophomores (25.19%), 130 juniors (24.81%), and 131 seniors (25.00%). Participants were recruited from three second-tier normal universities in central China, with nearly equal representation from each institution: 177 from University A (33.78%), 173 from University B (33.02%), and 174 from University C (33.21%). These institutions are comparable in terms of school level and students' college entrance scores, and they also have relatively consistent educational resources and teaching quality. Given the homogeneity of the participants' backgrounds, the findings can provide a reliable and general representation of students in second-tier normal universities within this region.

### 3.2 Data collection

Using a convenient sampling method, the questionnaires were administered online in January of 2025. Six English counselors from the three different universities were invited to distribute questionnaires to their students through the online platform Wenjuanxing. Before the survey, the counselors explained its purpose to the participants by WeChat, emphasizing that it is an academic research survey with purely academic significance. The responses were treated anonymously and confidentially. Participants were evenly distributed across three universities, with 200 participants from each university (200 × 3). Within each university, participants were also evenly distributed across four academic grades, with each grade contributing 50 individuals (50 × 4).

The electronic questionnaire was sent to the respondents by WeChat. Participants only need to tick the options that best describe themselves. Data collection spanned 1 week, yielding a total of 600 responses. After screening the data, 76 cases were invalid (e.g., those with significant missing data, multiple selections for a single question, or systematic repeating patterns in responses) and were removed prior to data analysis. Finally, 524 valid questionnaires were retained for analysis, resulting in an effective response rate of 87%.

### 3.3 Instrument

Based on the questionnaires from previous studies, the contents of the measurement items were modified to fit the purpose and context of this study. The original items were tailored for broad academic contexts, whereas this study focuses on EFL reading domain at the college level, therefore, modifications were necessary to enhance relevance and clarity in this context. Additionally, the modifications were made to align with cultural nuances. It was refined to make sure of the Chinese participants' understanding. Since the study was conducted in China, one of the researchers, who is bilingual in English and Chinese and has research experience in language education, first translated the items. A back-translation was then carried out by another bilingual researcher. Two Chinese experts, both associate professors specializing in motivation and second language acquisition, reviewed the original and back-translated versions to check semantic equivalence, cultural appropriateness, and clarity. Discrepancies were discussed and revised until consensus was reached, ensuring the accuracy of the scale translation. The questionnaire consists four scales covering students' perceptions of teacher support, student engagement, academic self-efficacy, and achievement goal orientation (see Appendix).

#### 3.3.1 Teacher support scale

To verify the research hypotheses, the teacher support scale was adapted from the *Teacher as a Social Context Questionnaire* (TASCQ; Belmont et al., 1992). The scale comprises three subscales—Teacher Autonomy Support (TAS), Teacher Relatedness Support (TRS), and Teacher Competence Support (TCS)—with three items each. Example items include “My English reading teacher offers me options for completing my assignments” (TAS), “My English reading teacher takes time to interact with me” (TRS), and “My English reading teacher ensures I fully understand before moving on” (TCS).

#### 3.3.2 Student engagement scale

The student engagement scale consists of behavioral engagement (BEG) and emotional engagement (EEG) adapted from Skinner et al. (2009), as well as cognitive engagement (CEG) adapted from Wolters (2004), which used the learning strategy items from the Metacognitive Strategies Questionnaire (MSQ), as major variables. Example items include “I focus during reading class” (BEG), “I try to connect what I'm learning to what I already know” (CEG), and “I task pleasure in learning new things in English reading class” (EEG).

#### 3.3.3 Student academic self-efficacy scale

The student academic self-efficacy scale was assessed using items from the Motivated Strategies for Learning Questionnaire (MSLQ) that was used to measure students' motivational beliefs and self-regulated learning (Pintrich and De Groot, 1990). A sample item is, for instance, “I am sure I can do an excellent job on the reading tasks”.

#### 3.3.4 Achievement goal orientation scale

The achievement goal orientation scale consists of mastery-approach goal orientation (MAG), performance-approach goal orientation (PApG), and performance-avoidance goal orientation (PAvG) as major variables, which was refined from the scale of Patterns of Adaptive Learning Survey (PALS) (Midgley et al., 1998). Sample items are, for instance, “It's important to me that I learn new knowledge in English reading class” (MAG); “One of my objectives is to demonstrate to others that I excel in my schoolwork” (PApG); and “I care about not appearing unintelligent in reading class” (PAvG).

The original scale was tailored to suit the Chinese university context and the specific focus on English reading. This process involved two types of adjustments: The wording of certain items slightly modified to align with Chinese linguistic conventions; anchored explicitly to the context of university-level EFL reading. Tables 1–4 illustrate the reliability and validity of the four scales in this study.

**Table 1:** Reliability, convergent validity, and the discriminant validity of the teacher support scale.

**Constructs**	**Item numbers**	**Reliability**	**Convergent validity**		**Discriminant validity**		
		**Cronbach's alpha**	**CR**	**AVE**	**TAS**	**TRS**	**TCS**
TAS	3	0.729	0.771	0.529	0.727		
TRS	3	0.599	0.761	0.515	0.553^***^	0.718	
TCS	3	0.797	0.800	0.572	0.623^***^	0.619^***^	0.756

TAS, Teacher Autonomy Support; TRS, Teacher Relatedness Support; TCS, Teacher Competence Support. Scale adapted from (Belmont et al. 1992). ^***^p < 0.001.

**Table 2:** Reliability, convergent validity, and the discriminant validity of the student engagement scale.

**Constructs**	**Item numbers**	**Reliability**	**Convergent validity**		**Discriminant validity**		
		**Cronbach's alpha**	**CR**	**AVE**	**TAS**	**TRS**	**TCS**
BEG	4	0.766	0.851	0.589	0.767		
CEG	3	0.742	0.790	0.557	0.532^***^	0.746	
EEG	3	0.807	0.815	0.595	0.456^***^	0.517^***^	0.772

BEG, Behavioral Engagement; CEG, Cognitive Engagement; EEG, Emotional Engagement. Scale adapted from (Skinner et al. 2009). ^***^p < 0.001.

**Table 3:** Reliability and convergent validity of the academic self-efficacy scale.

**Constructs**	**Item numbers**	**Reliability**	**Convergent validity**	
		**Cronbach's alpha**	**CR**	**AVE**
SE	6	0.782	0.885	0.563

SE, Academic self-efficacy. Scale adapted from Pintrich and De Groot (1990).

**Table 4:** Reliability, convergent validity, and the discriminant validity of the achievement goal orientation scale.

**Constructs**	**Item numbers**	**Reliability**	**Convergent validity**		**Discriminant validity**		
		**Cronbach's alpha**	**CR**	**AVE**	**TAS**	**TRS**	**TCS**
MAG	3	0.618	0.799	0.572	0.756		
PApG	4	0.744	0.821	0.535	0.453^***^	0.732	
PAvG	3	0.671	0.816	0.596	−0.455^***^	−0.368^***^	0.772

MAG, Mastery-approach Goal Orientation; PApG, Performance-approach Goal Orientation; PAvG, Performance-avoidance Goal Orientation. Scale adapted from (Midgley et al. 1998). ^***^p < 0.001.

### 3.4 Data analysis

The analyses were conducted using SPSS version 26.0 and AMOS version 24.0. The analysis began with an assessment of reliability and validity to ensure the robustness of the measurement model. To evaluate the suitability of the data for exploratory factor analysis (EFA), the Kaiser-Meyer-Olkin (KMO) measure and Bartlett's test of sphericity were employed. The KMO value of 0.855, 0.882, 0.904, and 0.822 for each construct, combined with a statistically significant Bartlett's test result (*p* < 0.001) confirmed the data's adequacy for EFA. The EFA model explained 69.522%, 69.410%, 63.444%, and 69.521% of the variance in teacher support, student engagement, academic self-efficacy, and achievement goal orientation, respectively, indicating a satisfactory level of variance extraction for each construct. In addition, the data were rotated using the Varimax rotation method to identify the correspondence between factors and research items. Table 5 presents the factor loadings, all of which exceeded the recommended threshold of 0.50, indicating a clear correspondence between each item and its respective factor.

**Table 5:** Exploratory factor analysis: rotated component matrix.

**Variable**	**Item**	**Component**		
		**1**	**2**	**3**
TS	TAS1		0.779	
	TAS2		0.782	
	TAS3		0.803	
	TRS4			0.826
	TRS5			0.791
	TRS6			0.728
	TCS7	0.881		
	TCS8	0.768		
	TCS9	0.801		
EG	BEG1	0.782		
	BEG2	0.809		
	BEG3	0.815		
	BEG4	0.785		
	CEG5			0.809
	CEG6			0.842
	CEG7			0.749
	EEG8		0.777	
	EEG9		0.834	
	EEG10		0.842	
SE	SE1	0.814		
	SE2	0.802		
	SE3	0.835		
	SE4	0.834		
	SE5	0.730		
	SE6	0.759		
GO	MAG1			0.736
	MAG2			0.861
	MAG3			0.830
	PApG4	0.759		
	PApG5	0.794		
	PApG6	0.780		
	PApG7	0.807		
	PAvG8		0.801	
	PAvG9		0.849	
	PAvG10		0.844	

To further assess construct validity, confirmatory factor analysis (CFA) was performed. Discriminant validity was established by comparing the square root of the average variance extracted (AVE) for each construct with the absolute values of its correlation coefficients with other constructs, thereby supporting discriminant validity. Convergent validity was confirmed, as all constructs demonstrated composite reliability (CR) values above the recommended threshold of 0.7 and AVE values exceeding 0.5, indicating strong internal consistency and reliability. Additionally, all factor loadings were statistically significant at the *p* < 0.05 level, further validating the measurement model.

Subsequent to the validity and reliability assessments, correlation analysis was conducted to examine the bivariate relationships among the study variables. Following this, the Structural Equation Modeling (SEM) approach was employed to test the proposed hypotheses. Model fit was evaluated using multiple indices. For the adequacy of the research model, a ratio of **x**^2^/df less than 3 (Tabachnick et al., 2013) and RMSEA below 0.08 indicate a good fit (MacCallum et al., 1996). The values of GFI, AGFI,NFI, TLI, and CFI greater than 0.90 are generally accepted (Hooper et al., 2008). A RMR below 0.05 is an acceptable fit (Rong, 2010). Additionally, the Harman single-factor test was used to examine Common Method Bias (CMB) in the data. All items in the study were subjected to factor analysis, and the variance explained by the first factor was 26.79%, which is below the critical threshold of 40%. This indicates that common method bias does not exist.

Further, the mediation effect of motivationally beliefs (academic self-efficacy) in the relationship between teacher support and student engagement was examined using the Bootstrapping resampling method. The study sample was resampled 5,000 times with a confidence level of 95%. If the obtained results do not include “0” within the confidence interval, it indicates that the mediation effect of the path is significant, confirming the correctness of the mediation effect test; otherwise, the mediation effect is not significant. Finally, the moderation effect of the motivational belief (achievement goal orientation) in the relationship between teacher support and student engagement was tested using Model 1 in the PROCESS plugin of SPSS software. Gender and grade, which showed significant differences in student engagement, were included as control variables. The independent variable and the moderator variable were mean-centered, and their product was used as an interaction term. The moderation effect was tested based on the significance of the interaction term.

## 4 Results

### 4.1 Relationships of the constructs

Table 6 shows the Pearson correlation matrix between the involved variables. The correlation coefficients reflect the linear correlation between the variables, providing a preliminary assessment of the hypotheses' reasonableness. It can be observed that there were significant correlations between the three forms of teacher support, academic self-efficacy, three dimensions of achievement goal orientation, as well as student engagement.

**Table 6:** Correlation analysis of teacher support and motivational beliefs.

**Variable**	**TAS**	**TRS**	**TCS**	**BEG**	**CEG**	**EEG**	**SE**	**MAG**	**PApG**	**PAvG**
TAS	1									
TRS	0.428^**^	1								
TCS	0.494^**^	0.413^**^	1							
BEG	0.385^**^	0.371^**^	0.408^**^	1						
CEG	0.414^**^	0.413^**^	0.453^**^	0.433^**^	1					
EEG	0.465^**^	0.411^**^	0.470^**^	0.381^**^	0.411^**^	1				
SE	0.471^**^	0.376^**^	0.488^**^	0.386^**^	0.426^**^	0.453^**^	1			
MAG	0.126^**^	0.175^**^	0.211^**^	0.128^**^	0.235^**^	0.153^**^	0.162^**^	1		
PApG	0.128^**^	0.176^**^	0.192^**^	0.128^**^	0.196^**^	0.192^**^	0.108^**^	0.390^**^	1	
PAvG	−0.110^**^	−0.126^**^	−0.142^**^	−0.163^**^	−0.196^**^	−0.158^**^	−0.142^**^	−0.370^**^	−0.304^**^	1

^*^p < 0.05; ^**^p < 0.01.

There were significant correlations between three forms of teacher support and student engagement, with correlation coefficients (*r*) ranging from 0.371 to 0.470. This range reflects a relatively strong positive relationship and corresponds to medium to large effect sizes based on Cohen's (1988) guidelines. These results indicate that higher levels of autonomy, relatedness, and competence support are associated with stronger behavioral, cognitive, and emotional engagement. Additionally, the negative *r* values between performance-avoidance goal and other variables indicate that these goals were negatively associated with teacher support and student engagement. The negative correlations suggest that performance-avoidance goals may weaken the positive relationships between teacher support and student engagement. Moreover, the correlation coefficient between academic self-efficacy and the three dimension of student engagement (*r* = 0.386, 0.426, 0.453), and those between academic self-efficacy and the three forms of teacher support (*r* = 0.471, 0.376, 0.488) were also significant, indicating a relatively strong positive relationship. These results confirm that students' academic self-efficacy exerts a medium-to large influence on both teacher support and student engagement.

### 4.2 Mediation effect of students' academic self-efficacy

#### 4.2.1 Model fit for the research model

**Figure 2:**
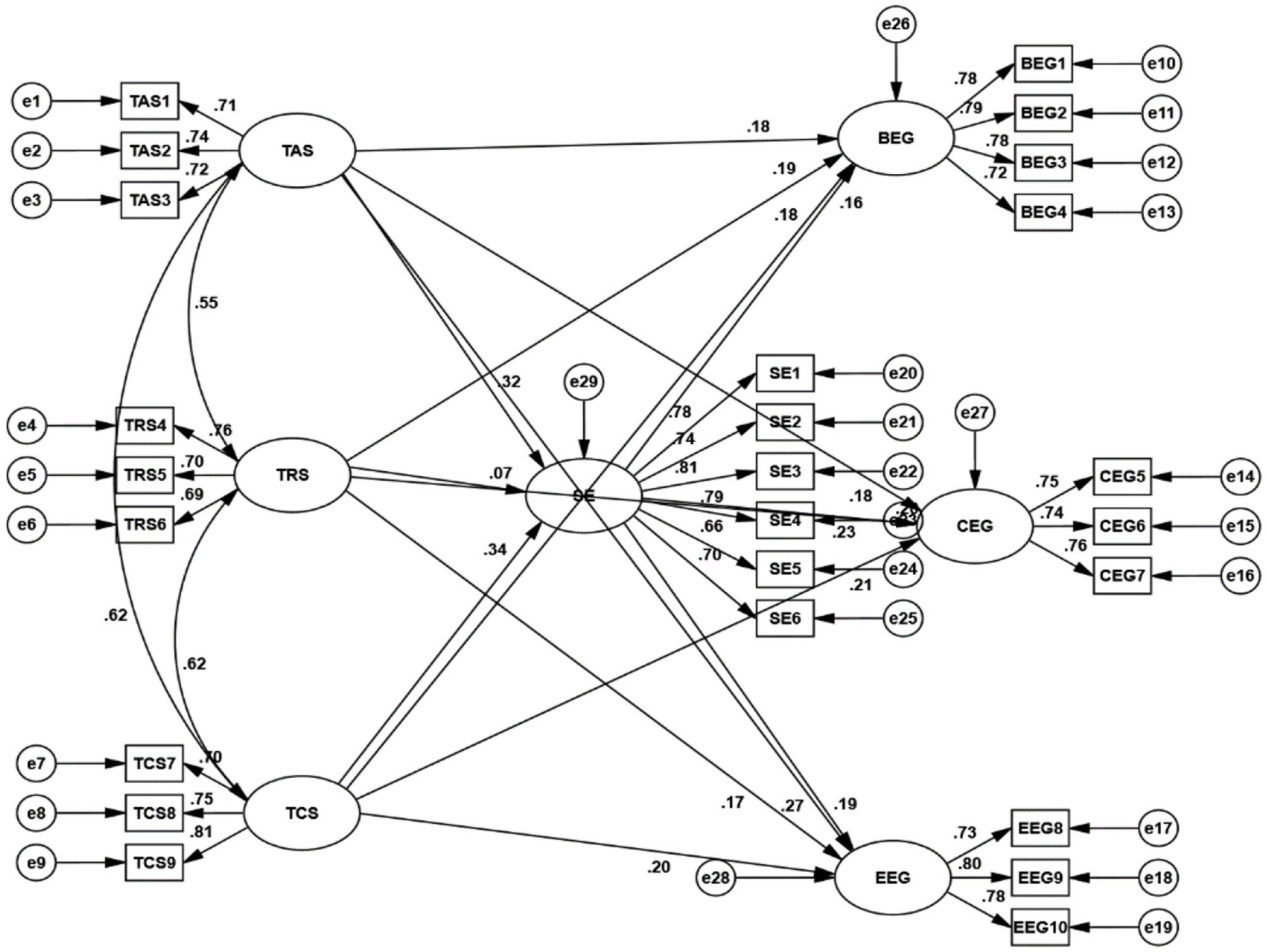
Research model and standardized estimates for SEM.

A path diagram was drawn as shown in Figure 2. The values of *x*^2^/df (1.340), RMSEA (0.026), RMR (0.034), GFI (0.950), AGFI (0.937), CFI (0.985), NFI (0.943), and TLI (0.982) collectively affirming the favorable fitness of the model.

#### 4.2.2 Mediation effect of academic self-efficacy

As shown in Table 7, the confidence intervals for the mediation paths TAS = >BEG (0.012 ~ 0.106), TAS = >CEG (0.023 ~ 0.127), TAS = >EEG (0.021 ~ 0.125), TCS = >BEG (0.011 ~ 0.117), TCS = >CEG (0.023~ 0.133), TCS = >EEG (0.021 ~ 0.130) do not include 0, indicating that SE as a mediator in the relationship between both teacher autonomy support and student engagement, as well as teacher competence support and student engagement. Although the indirect effects are modest in size (e.g., standardized β = 0.051 for TAS = > SE = > BEG), their effect size analyses across the three dimensions consistently demonstrate statistical significance. This provides empirical justification for testing mediation not only at the overall construct level but also at the dimension level. In particular, the findings confirm that academic self-efficacy contributes simultaneously to students' behavioural, cognitive, and emotional engagement, though the strength of effects varies slightly across dimensions. This dimension-level effect size analysis highlights that self-efficacy may be more strongly linked to cognitive and emotional engagement than to behavioural participation, thereby offering a more detailed understanding of the motivational pathway.

**Table 7:** Mediation effect of SE Between TS and EG.

**Path**	**Effect type**	**Estimate (β)**	**Lower**	**Upper**	***P***
TAS → SE → BEG	Direct	0.181	0.009	0.344	0.037
	Indirect	0.051	0.012	0.106	0.010
	Total	0.232	0.072	0.392	0.003
TAS → SE → CEG	Direct	0.179	0.007	0.333	0.042
	Indirect	0.062	0.023	0.127	0.002
	Total	0.241	0.087	0.384	0.004
TAS → SE → EEG	Direct	0.271	0.116	0.413	0.001
	Indirect	0.061	0.021	0.125	0.002
	Total	0.331	0.185	0.466	0.001
TRS → SE → BEG	Direct	0.191	0.019	0.343	0.033
	Indirect	0.011	−0.006	0.044	0.168
	Total	0.202	0.028	0.348	0.025
TRS → SE → CEG	Direct	0.226	0.060	0.378	0.008
	Indirect	0.014	−0.008	0.052	0.188
	Total	0.240	0.066	0.391	0.008
TRS → SE → EEG	Direct	0.172	0.013	0.313	0.036
	Indirect	0.014	−0.008	0.049	0.184
	Total	0.185	0.023	0.325	0.028
TCS → SE → BEG	Direct	0.185	0.003	0.362	0.046
	Indirect	0.054	0.011	0.117	0.011
	Total	0.238	0.059	0.403	0.012
TCS → SE → CEG	Direct	0.211	0.046	0.396	0.016
	Indirect	0.066	0.023	0.133	0.002
	Total	0.277	0.117	0.454	0.001
TCS → SE → EEG	Direct	0.196	0.039	0.362	0.017
	Indirect	0.065	0.021	0.130	0.002
	Total	0.260	0.106	0.422	0.001

### 4.3 Moderation effect of achievement goal orientation

#### 4.3.1 Moderation effect of mastery goal orientation (MAG)

**Figure 3:**
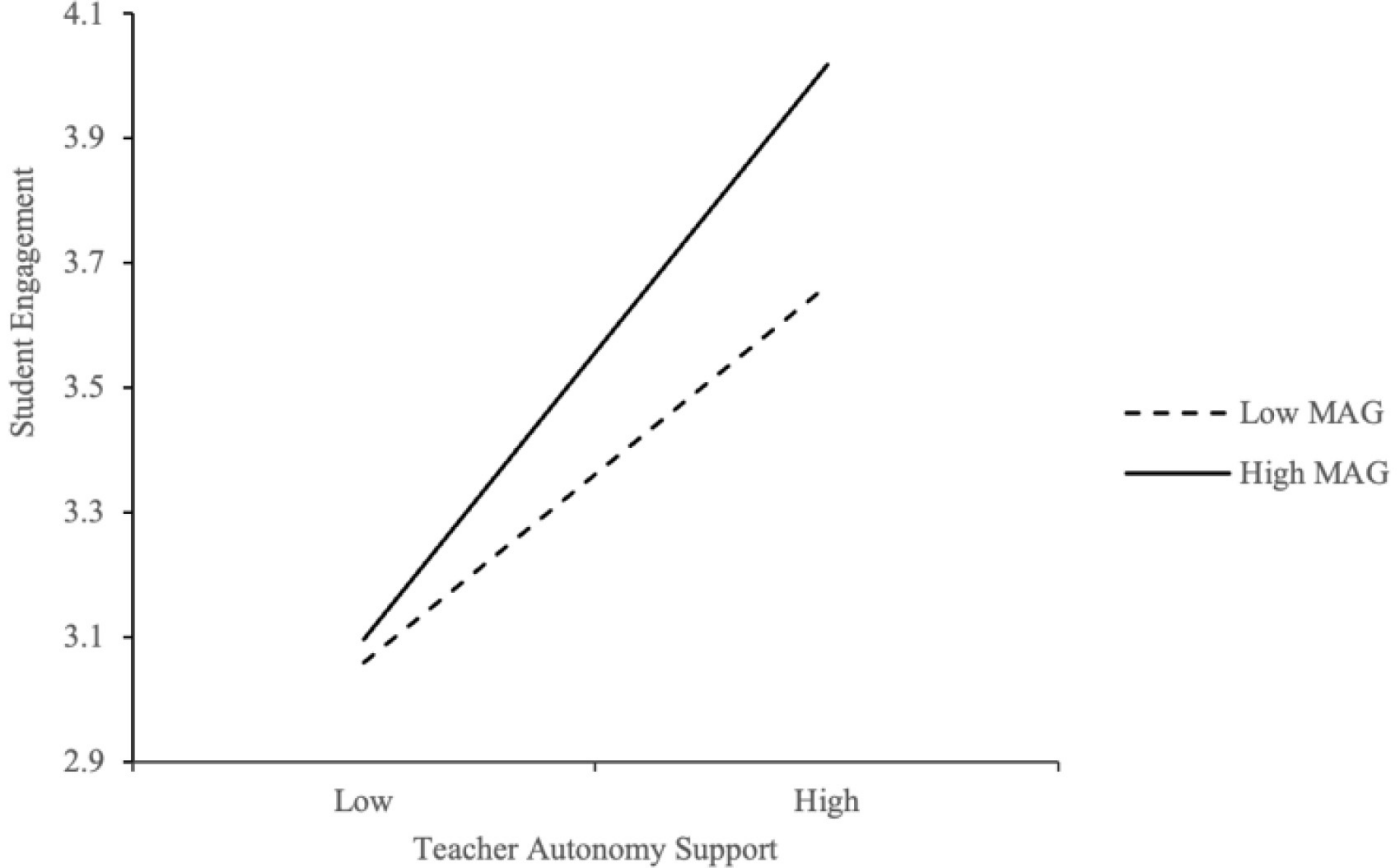
Moderation effect of MAG between TAS and EG.

To examine whether mastery goal orientation moderates the relationship between teacher autonomy support (TAS) and student engagement, hierarchical regression analyses were conducted. As shown in Table 8, the interaction term between TAS and mastery goal orientation was significant (*B* = 0.135, *p* < 0.01), suggesting a positive moderating effect. To visualize this interaction, simple slope analyses were plotted at ±1 SD from the mean (Figure 3). The results indicate that the positive association between TAS and student engagement was stronger among students with higher levels of mastery goal orientation. This indicates that mastery goal orientation functions as a moderator, such that autonomy support has a greater effect on engagement when students are strongly oriented toward mastery. The effect size (β = 0.135) confirms the statistical and practical significance of this moderation.

**Table 8:** Moderation effect of MAG.

**Tested moderating effect**	**Variable**	**B**	**S.E**.	**t**	***p***
Between TAS and EG	Constant	3.006	0.134	22.438	0.000^**^
	Gender	0.259	0.067	3.887	0.000^**^
	Academic year	−0.010	0.021	−0.492	0.623
	TAS	0.543	0.037	14.489	0.000^**^
	MAG	0.118	0.029	4.082	0.000^**^
	TAS^*^MAG	0.135	0.041	3.322	0.001^**^
	R	0.587			
	R2	0.345			
	F	F (5, 518) = 54.584, *p* = 0.000			
Between TRS and EG	Constant	2.932	0.139	21.130	0.000^**^
	Gender	0.249	0.069	3.604	0.000^**^
	Academic year	0.029	0.022	1.301	0.194
	TRS	0.531	0.041	12.870	0.000^**^
	MAG	0.100	0.030	3.296	0.001^**^
	TRS^*^MAG	0.047	0.048	0.980	0.328
	R	0.545			
	R2	0.297			
	F	F (5, 518) = 43.818, *p* = 0.000			
Between TCS and EG	Constant	3.078	0.135	22.856	0.000^**^
	Gender	0.208	0.067	3.113	0.002^**^
	Academic year	0.003	0.021	0.139	0.889
	TCS	0.493	0.037	13.476	0.000^**^
	MAG	0.081	0.029	2.757	0.006^**^
	TCS^*^MAG	−0.019	0.037	−0.499	0.618
	R	0.586			
	R2	0.343			
	F	F (5, 518) = 54.191, *p* = 0.000			

Dependent variable: student engagement.

^*^p < 0.05; ^**^p < 0.01.

This implies that mastery-oriented students benefit more from autonomy-supportive teaching practices as they exhibit greater engagement when given choice and responsibility in their learning. The effect size (β = 0.135) is practically meaningful, underscoring the importance of aligning autonomy support with students' self-referenced goals. Teachers may enhance engagement by promoting mastery goals alongside instructional autonomy.

#### 4.3.2 Moderation effect of performance-approach orientation (PApG)

**Figure 4:**
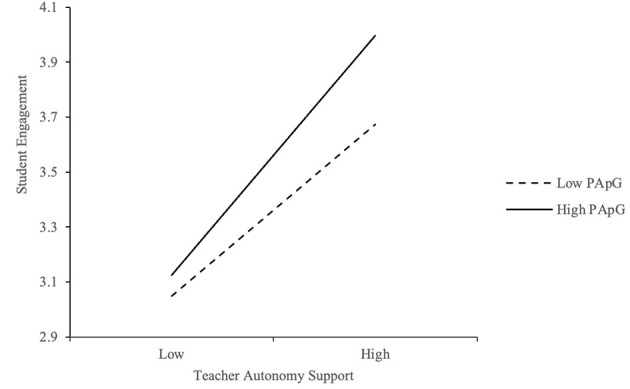
Moderation effect of PApG between TAS and EG.

**Figure 5:**
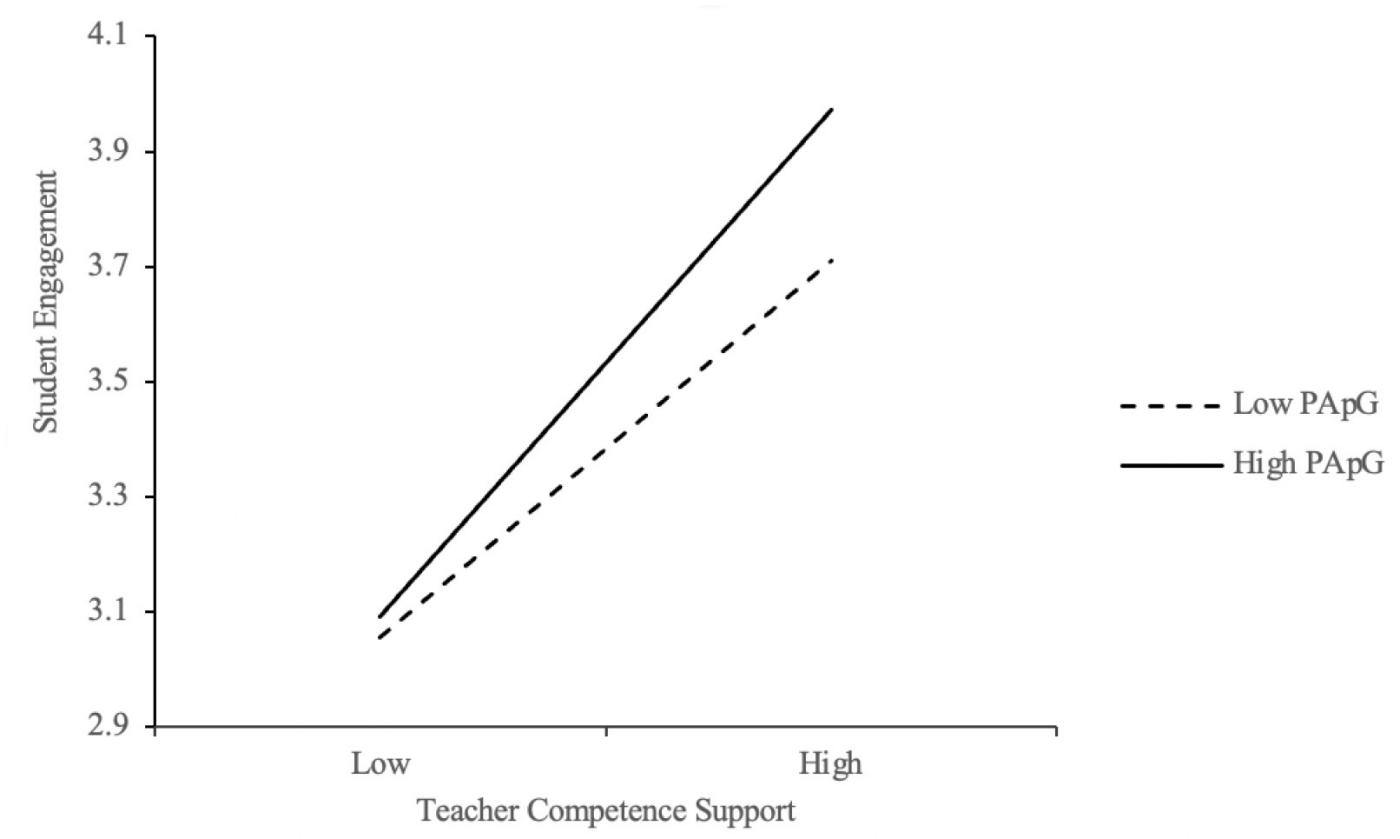
Moderation effect of PApG between TCS and EG.

To examine whether performance-approach goal orientation (PApG) moderates the relationship between teacher support and student engagement, interaction terms were tested. As shown in Table 9, the interactions between teacher autonomy support (TAS) and PApG (*B* = 0.108, *p* < 0.01), and between teacher competence support (TCS) and PApG (*B* = 0.097, *p* < 0.05) were both statistically significant, indicating positive moderation effects. Figures 4, 5 illustrate the interaction effects, with simple slopes plotted at high and low levels of PApG (±1 SD from the mean). The plots reveal that the positive relationships between TAS and engagement, and between TCS and engagement, were stronger among students with higher levels of performance-approach orientation. These students, motivated to demonstrate their competence, appear to benefit more from autonomy- and competence-supportive teaching practices. The effect sizes of these interactions (β = 0.108 for TAS × PApG; β = 0.097 for TCS × PApG) indicate small-to-moderate moderation effects, which are statistically meaningful in the educational context.

**Table 9:** Moderation effect of PApG.

**Tested moderating effect**	**Variable**	**B**	**S.E**.	**t**	***p***
Between TAS and EG	Constant	3.013	0.135	22.381	0.000^**^
	Gender	0.261	0.067	3.906	0.000^**^
	Academic year	−0.014	0.021	−0.659	0.510
	TAS	0.532	0.038	14.185	0.000^**^
	PApG	0.122	0.030	4.142	0.000^**^
	TAS^*^ PApG	0.108	0.040	2.691	0.007^**^
	R	0.584			
	R2	0.341			
	F	F (5, 518) = 53.607, *p* = 0.000			
Between TRS and EG	Constant	2.949	0.139	21.236	0.000^**^
	Gender	0.249	0.069	3.600	0.000^**^
	Academic year	0.022	0.022	1.002	0.317
	TRS	0.528	0.041	12.892	0.000^**^
	PApG	0.105	0.031	3.400	0.001^**^
	TRS^*^ PApG	0.045	0.045	1.011	0.312
	R	0.545			
	R2	0.297			
	F	F (5, 518) = 43.776, *p* = 0.000			
Between TCS and EG	Constant	3.073	0.133	23.063	0.000^**^
	Gender	0.211	0.066	3.174	0.002^**^
	Academic year	−0.002	0.021	−0.093	0.926
	TCS	0.534	0.036	14.739	0.000^**^
	PApG	0.092	0.030	3.110	0.002^**^
	TCS^*^ PApG	0.097	0.038	2.551	0.011^*^
	R	0.595			
	R2	0.354			
	F	F (5, 518) = 56.748, *p* = 0.000			

Dependent variable: student engagement.

^*^p < 0.05; ^**^p < 0.01.

#### 4.3.3 Moderation effect of performance-avoidance goal orientation (PAvG)

**Figure 6:**
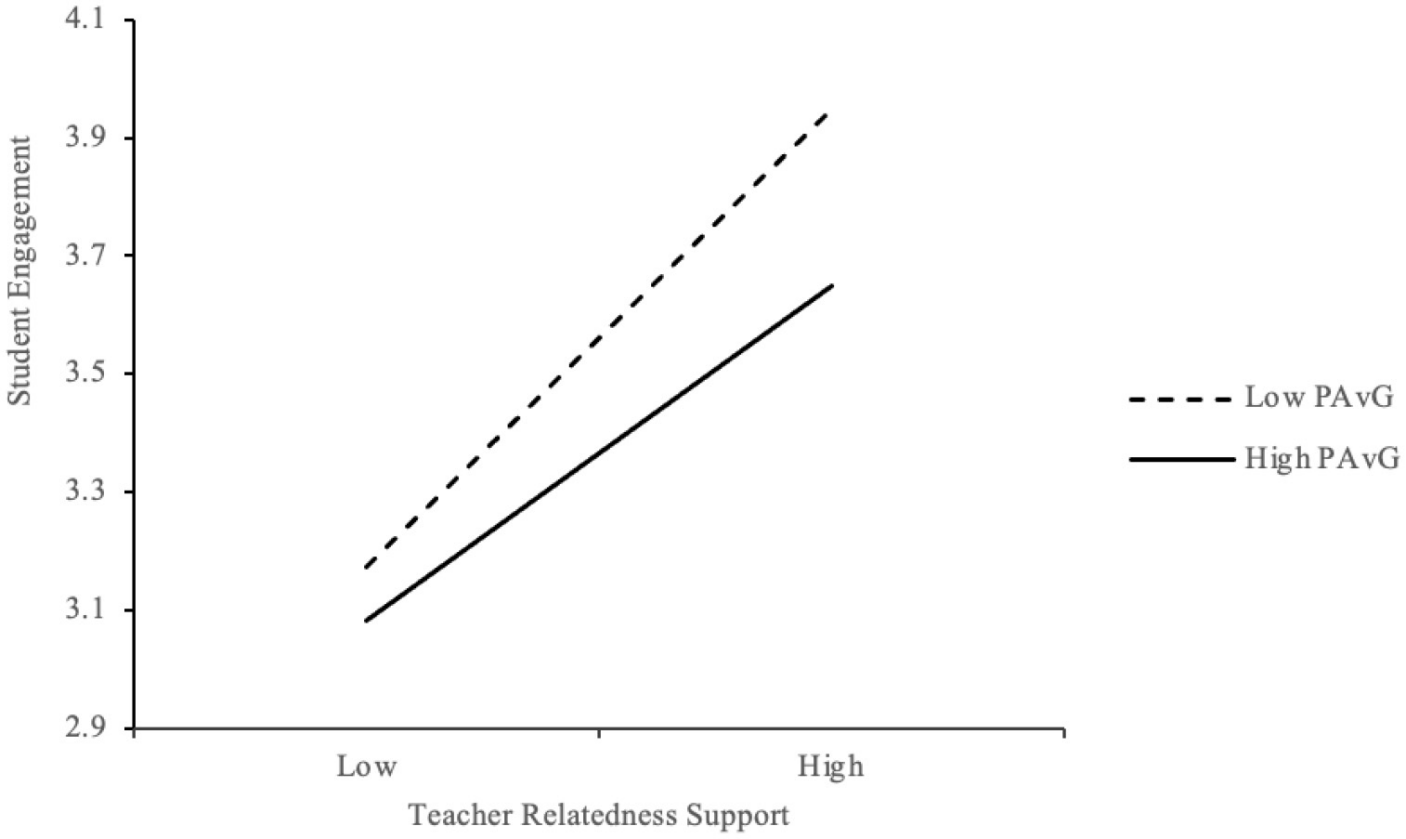
Moderation effect of PAvG between TRS and EG.

To examine the moderating role of performance-avoidance goal orientation (PAvG), interaction terms were tested. As shown in Table 10, the interaction between teacher relatedness support (TRS) and PAvG was significant (*B* = −0.092, *p* < 0.05), indicating a negative moderating effect. Figure 6 illustrates that the positive relationship between TRS and student engagement was weaker among students with higher PAvG. These students, motivated by fear of failure or negative evaluation, may be less receptive to emotional support, thereby limiting its impact on engagement.

**Table 10:** Moderation effect of PAvG.

**Tested moderating effect**	**Variable**	**B**	**S.E**.	***t***	***p***
Between TAS and EG	Constant	3.028	0.136	22.345	0.000^**^
	Gender	0.248	0.067	3.679	0.000^**^
	Academic year	−0.009	0.021	−0.414	0.679
	TAS	0.514	0.037	14.047	0.000^**^
	PAvG	−0.115	0.027	−4.318	0.000^**^
	TAS^*^ PAvG	−0.057	0.035	−1.644	0.101
	R	0.580			
	R2	0.336			
	F	F (5, 518) = 52.522, *p* = 0.000			
Between TRS and EG	Constant	2.974	0.138	21.548	0.000^**^
	Gender	0.228	0.069	3.310	0.001^**^
	Academic year	0.027	0.022	1.231	0.219
	TRS	0.539	0.040	13.429	0.000^**^
	PAvG	−0.107	0.027	−3.952	0.000^**^
	TRS^*^ PAvG	−0.092	0.043	−2.138	0.033^*^
	R	0.555			
	R2	0.308			
	F	F (5, 518) = 46.110, *p* = 0.000			
Between TCS and EG	Constant	3.105	0.135	23.066	0.000^**^
	Gender	0.193	0.067	2.886	0.004^**^
	Academic year	0.003	0.021	0.150	0.881
	TCS	0.499	0.035	14.294	0.000^**^
	PAvG	−0.100	0.026	−3.777	0.000^**^
	TCS^*^ PAvG	0.008	0.033	0.253	0.800
	R	0.593			
	R2	0.352			
	F	F (5, 518) = 56.189, *p* = 0.000			

Dependent variable: student engagement.

^*^p < 0.05; ^**^p < 0.01.

Although the effect size (β = −0.092) is modest, the pattern suggests that relational support alone may be insufficient for students with high avoidance tendencies. For these learners, EFL teachers should complement emotional support with scaffolded instruction that gradually builds confidence and reduces performance anxiety.

## 5 Discussion

From the perspective of teaching practice and teacher education, this study provides empirical evidence on the critical role of teacher support in shaping EFL learners' motivation and engagement for English reading. The findings reveal that among the three types of teacher support based on SDT, competence support exerts the strongest positive prediction on student engagement, followed by autonomy support, while relatedness support shows a relatively weaker impact.

Competence support is positively correlated with student engagement, which is aligned with previous studies (Hospel and Galand, 2016; Kiemer et al., 2018), consistent with cognitive load theory (CLT), which suggests that clear guidance reduces cognitive load, allowing students to focus on relevant information and facilitating learning. Competence support helps fulfill students' need for mastery, boosting intrinsic motivation. Interestingly, relatedness support has less predictive power for student engagement in college-level English reading classes, as university students, due to their developmental stage and increased autonomy, are less dependent on teachers for relational support. They seek more self-management and independent decision-making compared to younger students. Autonomy support (e.g., offering meaningful reading choices, encouraging self-regulated learning) also plays a key role, highlighting the need for teacher training to emphasize scaffolded autonomy—helping teachers strike a balance between structured guidance and student agency in reading tasks. These effects, while varying in strength, consistently reached medium-to-large effect sizes, highlighting their practical significance for instructional design. Therefore, teacher education programs should prioritize discipline-specific training in reading strategy instruction, discourse analysis, and differentiated scaffolding techniques.

These findings provide theoretical contributions to educational psychology by confirming that distinct types of teacher support influence engagement through motivational pathways, particularly academic self-efficacy and goal orientation. This underscores the value of contextualized psychological mechanisms in explaining how external instructional factors shape internal learner processes.

This study also suggests that teachers' instructional scaffolding, such as modeling metacognitive reading strategies and providing structured text analysis frameworks, in enhancing students' academic self-efficacy. Teachers can enrich reading experiences by employing various strategies (e.g., inferencing, predicting, visualizing, close reading, skimming, and multimodal reading), which not only foster interest in reading but also help students develop mastery-oriented reading habits, ultimately improving their reading proficiency and self-directed learning abilities. These findings call for a multidimensional approach to teacher development, ensuring that educators are equipped to provide strategic competence support, structured autonomy, and purposeful relatedness—all of which collectively enhance student motivation and engagement in English reading.

This study found significant correlations between academic self-efficacy, teacher support, and student engagement, aligning with Bandura's (1986) triadic causation model. Environment factors such as teacher support can influence personal factors such as academic self-efficacy and behavioral patterns such as learning engagement and vice versa. Specifically, teacher autonomy and competence support predicate student engagement through the mediation of self-efficacy in English reading classes, while relatedness support does not play a significant role. It aligns with Ruzek et al.'s (2016) finding that self-efficacy beliefs are more effectively shaped by instructionally-supported interactions rather than emotionally-supported teacher-student relationships. The mediation pathways, although modest in size, showed small-to-moderate effect sizes, suggesting that even indirect effects can meaningfully sustain student engagement.

To further develop students' academic self-efficacy in English reading, teachers should ensure that tasks with students' ability levels. Appropriately challenging tasks enhance focus and engagement, while overly difficult tasks may undermine motives and self-belief. By designing instruction that aligns with English learners' Zone of Proximal Development (ZPDs), teachers can support optimal growth within student' capability range. Differentiated reading tasks tailed to proficiency levels and opportunities for autonomy allow learners to experience genuine accomplishment. Moreover, setting clear reading goals, providing constructive and timely feedback, and gradually increasing task difficulty can steadily build confidence. When students struggle, scaffolding support is essential for maintaining motivation and progress.

Achievement goal orientations influence how teacher support translates into student engagement in English reading. Mastery goals strengthen the relationship between autonomy support and engagement, as students oriented toward growth are more receptive to autonomy. Performance-approach goals also enhance the effects of both autonomy and competence support since such students are motivated to achieve through effort and thus benefit from structured teacher guidance and encouragement. Conversely, performance-avoidance goals weaken the relationship between relatedness support and engagement because students aiming to avoid failure are less likely to seek help, which lowers their motivation and involvement. The moderation effects, although varying in size, ranged from small to moderate, highlighting that the strength of teacher support–engagement links depends on students' motivational orientations.

These findings underscore the importance of integrating motivational perspectives within educational psychology to capture individual differences in learner responses. Overall, the study demonstrates how external teacher support interacts with internal psychological factors to foster engagement in second language reading. It emphasizes that teacher education should focus on equipping teachers with the ability to provide targeted competence support, structured autonomy, and meaningful relational support—together creating conditions that nurture both engagement, academic self-efficacy, and goal orientation in reading.

## 6 Conclusion

This study extends previous research in SDT by exploring the prediction of teacher support for students' motivational beliefs and student engagement in Chinese EFL college classrooms. However, it has several limitations. First, the study used a cross-sectional design and relied solely on student self-reports, which may limit the accuracy of understanding the full scope of how teacher support predicts motivation. Future research should incorporate teacher reports to offer a more thorough understanding of the connections between teacher support, personal motivations, and student engagement. Additionally, the survey analysis was based on how students perceived their existing classes, perhaps a deeper story could be explained if there was an experimental intervention with specific teacher support strategies being used in the classes to see the specific effects on certain types of student engagement. Moreover, our moderation analysis only examined the main effect of teacher support on student engagement. However, reporting the relationship between the moderator (GO) and the independent variable (TS) would be developed to make a full moderation analysis in the future study.

This study sheds light on the differentiated effects of teacher support on student motivation and engagement in Chinese university EFL reading classes. By incorporating effect size analyses, the study shows that these relationships range from small to large in magnitude, highlighting not only statistical significance but also practical importance. Overall, the findings provide meaningful implications for designing teacher education programs and classroom practices that strengthen student engagement in EFL reading by supporting learners' motivation and confidence.

## Data Availability

The original contributions presented in the study are included in the article/Supplementary material, further inquiries can be directed to the corresponding author.

## Appendix

**Table A1:** Teacher support scale.

**Cons**.	**Items**	
TS	TAS1	My English reading teacher offers me options for completing my assignments.
	TAS2	My English reading teacher is always telling me what to do.
	TAS3	My English reading teacher values my ideas.
	TRS4	My English reading teacher is fond of me.
	TRS5	My English reading teacher understands me.
	TRS6	My English reading teacher takes time to interact with me.
	TCS7	My English reading teacher doesn't make his expectations for me clear.
	TCS8	My English reading teacher teaches me how to solve problems on my own.
	TCS9	My English reading teacher ensures I fully understand before moving on.

TS, Teacher Support; TAS, Teacher Autonomy Support; TRS, Teacher Relatedness Support; TCS, Teacher Competence Support.

**Table A2:** Student engagement scale.

**Cons**.	**Items**	
EG	BEG1	I put in my best effort in English reading class.
	BEG2 BEG3	I take part in reading class discussion. I focus during reading class.
	BEG4	I listen attentively when I am in reading class.
	CEG5	I try to relate what I am learning to my own experiences.
	CEG6	I try to organize all different ideas and make them coherent when I study for English reading class.
	CEG7	I try to connect what I'm learning to what I already know.
	EEG8	When we work on something, I feel interested.
	EEG9	English reading class is fun.
	EEG10	I task pleasure in learning new things in English reading class.

EG, Student Engagement; BEG, Behavioral Engagement; CEG, Cognitive Engagement; EEG, Emotional Engagement.

**Table A3:** Academic self-efficacy scale.

**Cons**.	**Items**	
SE	SE1	I'm certain I can understand the ideas taught in English reading course.
	SE2	I am sure I can do an excellent job on the reading tasks.
	SE3	1 think I will receive a good grade in English reading class.
	SE4	My study skills are excellent compared with others in reading class.
	SE5	Compared with others in English reading class, I think I'm a good student.
	SE6	I know that I will be able to learn the material for reading class.

SE, Academic self-efficacy.

**Table A4:** Achievement goal orientation scale.

**Cons**.	**Items**	
GO	MAG1	It's important to me that I learn new knowledge in English class.
	MAG2	One of my goals in class is to learn as much as possible.
	MAG3	It's essential for me to have a deep understanding of my coursework.
	PApG4	I care about having my classmates view me as competent in my schoolwork.
	PApG5	One of my objectives is to demonstrate to others that I excel in my schoolwork.
	PApG6 PApG7	One of my aims is to prove to others that I find classwork is effortless for me. One of my objectives is to appear more intelligent compared to my classmates.
	PAvG8	I care about not appearing unintelligent in class.
	PAvG9	I want to make sure that my teacher doesn't see me as knowing less than my classmates.
	PAvG10	One of my aims in class is to avoid seeming like I am having trouble with the work.

GO, Achievement Goal Orientation; MAG, Mastery-approach Goal Orientation; PApG, Performance-approach Goal Orientation; PAvG, Performance-avoidance Goal Orientation.
